# Fortification of Pectin/Blackberry Hydrogels with Apple Fibers: Effect on Phenolics, Antioxidant Activity and Inhibition of α-Glucosidase

**DOI:** 10.3390/antiox11081459

**Published:** 2022-07-27

**Authors:** Mirela Kopjar, Ina Ćorković, Ivana Buljeta, Josip Šimunović, Anita Pichler

**Affiliations:** 1 Faculty of Food Technology, Josip Juraj Strossmayer University, F. Kuhača 18, 31000 Osijek, Croatia; ina.corkovic@ptfos.hr (I.Ć.); ivana.buljeta@ptfos.hr (I.B.); anita.pichler@ptfos.hr (A.P.); 2 Department of Food, Bioprocessing and Nutrition Sciences, North Carolina State University, Raleigh, NC 27695-7624, USA; simun@ncsu.edu

**Keywords:** pectin/blackberry hydrogels, apple fiber, anthocyanins, antioxidant activity, α-glucosidase

## Abstract

The objective of this study was to prepare hydrogels based on pectin and blackberry juice and additionally to fortify those hydrogels with apple fiber. For that purpose, two types of pectin (low methoxylated and high methoxylated) were used, and fortification was conducted with the addition of 10% of apple fiber. The hydrogels were evaluated for phenolic compounds, antioxidant activity and inhibition of α-glucosidase. In addition, the stability of these parameters after 8 months of storage was evaluated. Pectin type and addition of apple fiber had an impact on investigated parameters. Low methoxylated pectin hydrogels had a higher concentration of anthocyanins than high methoxylated pectin hydrogels, while the addition of apple fibers caused a decrease in anthocyanin content. However, fortified hydrogels had higher antioxidant activity due to the presence of phenolics from apple fibers. The results showed that anthocyanins were more favorable in inhibiting α-glucosidase because samples with higher anthocyanins concentration had lower IC_50_ values. Obtained hydrogels can be used as intermediate products or ingredients (like fruit fillings or spreads) for the improvement or development of novel food products to increase their fiber content and antioxidant potential.

## 1. Introduction

More than ever, nutritional guidelines are highlighting the importance of the consumption of fruits and their products in everyday life. In general, berry fruits are widely recommended for their high content of valuable bioactive compounds, especially flavonoids, phenolic acids, tannins, and anthocyanins. Those bioactive compounds possess a high antioxidant potential, and they can individually and/or synergistically help protect our organisms against cardiovascular disorders, cancer, inflammation, obesity, and other chronic diseases. Blackberries are very popular among consumers, known for their high content of bioactive compounds, especially anthocyanins and ellagitannins [1,2,3], which are responsible for their positive impact on health [4,5]. It is known that these compounds inhibit α-glucosidase and consequently control starch digestion, reduce hyperglycemia, and regulate blood glucose levels. Their inhibitory activity has been strongly related to their structure [6]. Binding interactions between α-glucosidase and phenolics have been studied using different methods such as IC_50_ value, inhibition kinetics, molecular docking, and others. Phenolics, in the form of extracts and as pure substances, are being studied as potential alternatives to commercially available medicines for hyperglycemia because their application could reduce the likelihood of the occurrence of side effects [7].

Due to the high and rapid perishability of berries, as well as their very high nutritional value, bioactive compounds must be transferred from berries into products. Traditional products would include juices, jams, jellies, and canned fruits. However, there is a need for the development of novel formulations that would protect bioactive components and improve their functionality. Those novel formulations can be used as intermediate products or ingredients (like fruit fillings or spreads) for the improvement or development of novel food products.

Like the phenolic compounds, dietary fibers are also well known for their positive influence on our health. They have an essential role in intestinal health and additionally, they are associated with a reduction of hypercholesterolemia and modification of the glycemic response [8]. They are defined as “the remnants of the edible part of plants or analogous carbohydrates that are resistant to digestion and absorption in the human small intestine, with complete or partial fermentation in the large intestine” [9]. Research on interactions between dietary fibers and phenolics as well as the application of different fibers as delivery systems for phenolic compounds are emerging trends [10,11,12,13,14,15,16,17,18,19,20,21,22,23,24]. We selected apple fibers for the fortification of hydrogels with fibers. It is known that apple fibers also contain bioactive compounds such as flavonoids, phenolics and carotenoids, so consequently they have been considered as a source of better-quality dietary fiber [25,26]. Studies on the application of fibers mainly include the addition of dietary fiber to flour, meat, and dairy products, or its use as additives [27]. On the other hand, there is a challenge to the food industry to incorporate fiber in traditional products or to develop new fiber-rich products [28]. With this perspective, we selected fortification of pectin-based hydrogels with fibers. Due to the imperative of safe consumption, hydrogels for food applications are based on polymers that are present in nature [29] and they can have a considerable impact on the retention of phenolic and flavor compounds [30]. Pectin has a quite important role in the food industry due to its application in a variety of different products. Its functional properties, such as gelling, thickening and emulsification, are the most important factors for its wide application. Those properties depend on its chemical structure, especially carboxyl functional groups that determine its ability to interact with other compounds through coordination, electrophilic addition, esterification, transesterification reactions [31]. Additionally, those interactions can improve the retention and stability of phenolic compounds [11,12,32,33,34].

The aim of this study was to prepare pectin/blackberry hydrogels unfortified or fortified with the addition of apple fibers. Obtained hydrogels were investigated for the stability of anthocyanins during the preparation of hydrogels as well as after 8 months of storage, depending on the composition of formulations. In addition, the antioxidant potential of prepared hydrogels and inhibition of α-glucosidase were monitored.

## 2. Materials and Methods

### 2.1. Chemicals

The apple fiber powder was supplied by Biesterfeld AG (Zagreb, Croatia) and both types of pectin were the product of CP Kelco (Atlanta, GA, USA). The 2,2-diphenyl-1-picrylhydrazyl (DPPH), 2,2-azino-bis(3-ethylbenzothiazoline-6-sulfonic acid) diammonium salt (ABTS), trolox, 4-(dimethylamino)-cinnamaldehyde (DMAC), chlorogenic, caffeic, p-coumaric, gallic and ellagic acids, rutin, quercetin, phloretin and phlorizin were purchased from Sigma-Aldrich (St. Louis, MO, USA). The α-glucosidase (from *Saccharomyces cerevisiae*) and acarbose were obtained from Sigma-Aldrich (St. Louis, MO, USA). The 4-nitrophenyl-α-D-glucopyranoside was obtained from Alfa Aesar (Kandel, Germany) and potassium dihydrogen phosphate was from BDH Prolabo (Poole, UK). Potassium persulfate, Folin-Chiocalteu reagent and sodium carbonate were obtained from Kemika (Zagreb, Croatia). Neocuproine, 2,4,6-tri(2-pyridyl)-s-triazine (TPTZ) and copper (II) chloride were purchased from Acros Organics (Geel, Belgium). Methanol (HPLC grade) was from J.T. Baker (Deventer, The Netherlands) and orthophosphoric acid (HPLC grade > 85%) was from Fisher Scientific (Loughborough, UK). Iron (III) chloride hexahydrate, sodium acetate, ethanol, ammonium acetate and starch were bought from Gram-mol (Zagreb, Croatia). Cyanidin 3-glucoside was from Extrasynthese (Genay, France).

### 2.2. Formulation of Hydrogels

The juice was obtained by pressing blackberry fruits and filtering the resulting mass. First, blackberry juice was heated to 70 °C on a magnetic stirrer. In the next step, 3% of low methoxylated or high methoxylated pectin and 10% of CaCl_2_ solution (100 mM) were added. For each type of pectin, one sample was enriched with the addition of apple fiber (10%). After homogenization for 20 min, the hot mixtures were poured into preheated glass jars. Hydration was completed overnight and then the samples were analyzed. The other set of samples was stored for 8 months at room temperature and in the presence of light.

### 2.3. Extraction of Hydrogels

Before the HPLC analysis and evaluation of antioxidant activity, extraction was carried out. For the extraction of hydrogels, 3 g of sample was mixed with 30 mL of acidified methanol (HCl:methanol ratio was 1:99). The obtained mixture was well homogenized and left for 24 h. After that time, the mixture was filtered to obtain the clear extract.

### 2.4. Spectrophotometric Determination of Total Phenolics and Proanthocyanidins

A slightly modified Folin–Ciocalteu method was used to determine the total phenolics. This method had been previously described by Singleton and Rossi [35]. First, 0.2 mL of extract was added to the glass tube followed by the addition of 1.8 mL of deionized water, 10 mL of Folin–Ciocalteu reagent (1:10) and 8 mL of sodium carbonate (7.5%). Prepared mixtures were kept in the dark. After 120 min, the absorbance of the samples was measured at 765 nm using a spectrophotometer (Cary 60, UV-VIS, Agilent Technologies, Santa Clara, CA, USA). Measurements were conducted in triplicates. The obtained values were interpolated on a gallic acid calibration curve and the results were expressed as g of gallic acid equivalents per kg of hydrogel or per liter for juice (g GAE/kg or g GAE/L).

Total proanthocyanidins were determined using a method by Prior et al. [36]. Briefly, 0.1 mL of extract was mixed with 0.4 mL of acidified ethanol and 1 mL of 4-dimethyl-amino-cinnamaldehyde solution. Mixtures were kept in the dark for 30 min and absorbance was measured at 640 nm. Measurements were performed in triplicate. Calibration curve was created for procyanidin B2 and total proanthocyanidins were expressed as mg of procyanidin B2 equivalent per kg of hydrogel or per liter for juice (mg B2E/kg or mg B2E/L).

### 2.5. HPLC Analysis for Identification and Quantification of Phenolics

First, extracts were filtered through 0.2 µm PTFE membranes. For the evaluation of phenolics, HPLC (High-performance liquid chromatography) system 1260 Infinity II (Agilent technology, Santa Clara, CA, USA) was applied. The method used was previously described by Ivić et al. [37]. The system was equipped with a quaternary pump, a vial sampler, DAD detector and a Poroshell 120 EC C-18 column (4.6 × 100 mm, 2.7 µm). Calibration curves were performed for cyanidin-3-*O*-glucoside, quercetin, chlorogenic acid, ellagic acid, rutin, caffeic acid, gallic acid, phloretin and phlorizin hydrate. Cyanidin-3-*O*-dioxalylglucoside was tentatively identified by comparing its peak spectrum with standard and literature data and was quantified using cyanidin-3-glucoside. The UV/Vis spectra were recorded in the range of 190 to 600 nm and used for identification of peaks.

### 2.6. Determination of Antioxidant Activity

For the evaluation of antioxidant activity, FRAP, CUPRAC, DPPH and ABTS methods were used. The FRAP antioxidant capacity of samples was determined according to the Benzie and Strain method [38] with some modifications. Cupric reducing antioxidant capacity assay was carried out according to the method of Apak et al. [39]. The ABTS assay followed the method of Arnao et al. [40] with some modifications. The antioxidant activity of the samples was also determined by the radical scavenging activity method using 2,2-diphenyl-1-picrylhydrazyl radical (DPPH) [41].

Antioxidant activities evaluated by the mentioned methods were calculated from the calibration curve with Trolox as standard and expressed as µmol TE/100 g. For each method, all measurements were performed in triplicates and for the blank, the extract was replaced with water.

### 2.7. Estimation of Synergism

For evaluation of additive, synergistic or antagonistic effects, experimental values of antioxidant activity of fortified hydrogels were compared to the theoretical ones. Theoretical antioxidant activity was obtained by calculating the sum of the antioxidant activity of blackberry juice and the antioxidant activity of apple fiber. These values were compared with experimentally obtained antioxidant activities of fortified hydrogels. Higher experimental values of fortified hydrogels than theoretical ones indicate a potentially synergistic effect. Lower experimental values of fortified hydrogels than theoretical ones indicate a potentially antagonistic effect. The numerically equal experimental and theoretical values of fortified hydrogels indicate an additive effect.

### 2.8. Determination of α-Glucosidase Inhibition

Inhibition of α-glucosidase was determined spectrophotometrically using the method previously described in the study by Granados-Guzman et al. [42] with slight modifications. Solutions of α-glucosidase, substrate (4-nitro-phenyl-α-D-glucopyranoside), different concentrations of phenolic extracts and acarbose were prepared in phosphate buffer (0.1 M, pH 6.8). In this experiment, acarbose as a well-known inhibitor was used as a positive control. To determine the inhibition of α-glucosidase, it was necessary to prepare a control blank, negative control, inhibition blank and inhibition. To prepare an inhibition and inhibition blank, 165 µL of different phenolics concentrations of tested sample were mixed with 85 µL of substrate (0.02 mg/mL) and incubated at 37 °C for 5 min. To the control blank and negative control, phosphate buffer was added instead of the sample. To the negative control and inhibition, 85 µL of α-glucosidase (1.93 µg/mL) was added after incubation and after the addition of the enzyme, incubation was repeated at the same temperature for 17.5 min. In the control blank and inhibition blank, the enzyme was replaced with phosphate buffer. The reaction was stopped after adding 665 µL of sodium carbonate solution (0.1 M). Each sample was analyzed in triplicate and absorbance was measured at 405 nm using a spectrophotometer (Cary 60, UV-VIS, Agilent Technologies, Santa Clara, CA, USA). Percentages of inhibition were calculated using the formula (%):

$$\%=\frac{\left(\left({\mathrm{Abs}}_{\mathrm{negative}\;\mathrm{control}}{-\mathrm{Abs}}_{\mathrm{control}\;\mathrm{blank}}\right)-\left({\mathrm{Abs}}_{\mathrm{inhibition}}{-\mathrm{Abs}}_{\mathrm{inhibiton}\;\mathrm{blank}}\right)\right)}{\left({\mathrm{Abs}}_{\mathrm{negative}\;\mathrm{control}}{-\mathrm{Abs}}_{\mathrm{control}\;\mathrm{blank}}\right)}\times 100$$

Concentrations of inhibitors that exhibited 50% inhibition of enzyme activity (IC_50_) were determined using a graphical method and plots of inhibition percentage *versus* log inhibitor concentration. The IC_50_ values were then calculated by non-linear regression analysis from the mean inhibitory values.

### 2.9. Statistical Analysis

Comparison of formulated complexes was conducted by analysis of variance (ANOVA) and Fisher’s least significant difference (LSD) with the significance defined at *p* < 0.05. Furthermore, principal component analysis (PCA) on volatile compounds was conducted. The software program STATISTICA 13.1 (StatSoft Inc., Tulsa, OK, USA) was used for statistical analyses. The results were presented as the mean values ± standard deviation.

## 3. Results and Discussion

### 3.1. Phenolic Compounds

The results for the concentration of total phenolics and proanthocyanidins determined in blackberry juice, apple fiber and hydrogels after preparation, as well as after storage, are presented in Table 1. Both unfortified hydrogels had lower concentrations of total phenolics than initial blackberry juice (62.59 mg GAE/100 g), i.e., a 28% degradation rate of phenolics during hydrogels preparation was observed. Fortification of hydrogels resulted in the increase of total phenolics in comparison to blackberry juice as apple fiber contained its own phenolics. Comparing different hydrogels, it was observed that samples fortified with apple fiber contained higher concentrations of total phenolics after preparation and after storage. In the case of hydrogels enriched with apple fiber, concentrations of total phenolics ranged from 97.60 mg GAE/100 g (LMP + AF) to 109.8 mg GAE/100 g (HMP + AF) and for the unfortified hydrogels, concentration of total phenolics was around 45 mg GAE/100 g. For the hydrogels with apple fiber, it was concluded that the type of pectin affected the concentration of total phenolics, and HMP + AF hydrogel showed a higher concentration of total phenolics than LMP + AF hydrogel. In the case of unfortified hydrogels, there was no significant difference between LMP and HMP samples. After 8 months of storage at room temperature under light, the concentrations of phenolics were reduced and ranged from 39.20 mg GAE/100 g to 79.01 mg GAE/100 g. Stored samples showed a similar trend as samples after preparation; unfortified samples had lower concentrations of total phenolics than those fortified with apple fiber. The type of pectin affected values for total phenolics of stored samples and LMP hydrogel contained higher concentrations of total phenolics (45.10 mg GAE/100 g) than HMP hydrogel (39.20 mg GAE/100 g), and LMP + AF hydrogel had higher concentration of total phenolics (79.01 mg GAE/100 g) than HMP + AF hydrogel (75.80 mg GAE/100 g). Pectin has a high affinity toward polyphenols compared to other polysaccharides. Interactions between polysaccharides and polyphenols depend on the structure and morphology of polysaccharides as well as on molecular weight, functional groups, and conformational changes of polyphenols [43]. For that reason, in addition to determining the concentrations of total phenolics, it is useful to know the concentrations of individual polyphenols in the matrix for better understanding of these interactions.

From the results for total proanthocyanidins content, it was concluded that apple fiber served as a great source of these bioactive compounds. Similar findings were observed in the study by Kołodziejczyk et al. [44]. Samples fortified with apple fiber had significantly higher concentrations of proanthocyanidins (98.55 µg/g and 110.20 µg/g for LMP + AF and HMP + AF hydrogel, respectively) than unfortified samples (12.54 µg/g and 14.66 µg/g for LMP and HMP hydrogel, respectively). These samples contained higher concentrations of proanthocyanidins even after storage (34.88 µg/g and 28.42 µg/g for LMP + AF and HMP + AF hydrogel, respectively). Interactions between plant cell walls polysaccharides and proanthocyanidins have been attracting the attention of the scientific community since 2000. The highest affinity of proanthocyanidins for cell wall components (cellulose framework, pectin matrix and cross-linked glycoproteins) was determined for pectin. This was explained by the formation of hydrophobic pockets, which encapsulate proanthocyanidins and form a three-dimensional gel-like network [45]. Therefore, it was concluded that interactions between pectin and proanthocyanidins influenced their retention in our samples. However, these interactions depend on the molecular weight, structure, degree of polymerization and concentration of proanthocyanidins, as well as on the physical and chemical composition of the polysaccharides [45]. The results obtained in the present study showed that the degree of methylation of pectin had an impact on the binding of proanthocyanidins as HMP + AF hydrogel had a higher amount of proanthocyanidins than LMP + AF hydrogel after preparation. These results were in agreement with those previously published in literature which have reported that highly methylated galacturonic acid chains from pectin have a high affinity to procyanidins through hydrophobic interactions, where methyl groups of pectic fractions create interactions with dihydropyran heterocycles of procyanidins [45]. On the other hand, after storage, the trend was reversed and LMP + AF hydrogel had higher concentrations of proanthocyanidins compared to HMP + AF hydrogel.

Concentration values for individual phenolic compounds and anthocyanins detected in blackberry juice and apple fibers are presented in Table 2, while the results for hydrogels are presented in Table 3. Two anthocyanins, very important pigments responsible for blackberry color, were detected in blackberry juice as well as in hydrogels, namely cyanidin-3-*O*-glucoside and cyanidin-3-*O*-dioxalylglucoside. The type of pectin used for hydrogels preparation as well as the addition of apple fiber had an impact on both anthocyanins. The LMP hydrogel had a higher amount of both anthocyanins (72.89 mg/kg and 27.09 mg/kg, respectively) than HMP hydrogel (70.50 mg/kg and 26.01 mg/kg, respectively). The addition of apple fiber negatively affected anthocyanins, probably due to interactions of apple fibers components with them. Their amounts in fortified hydrogels were 57.15 mg/kg and 20.56 mg/kg, respectively, for LMP hydrogel, and 55.61 mg/kg and 20.77 mg/kg, respectively, for HMP hydrogel. Calculating the retention of anthocyanins during the preparation of hydrogels, it was concluded that both anthocyanins were retained in quite high amounts in correspondence to blackberry juice, over 93.5%. However, higher retention of both anthocyanins was observed for LMP hydrogels. In blackberry juice, ellagic acid, chlorogenic acid, caffeic acid and gallic acid were detected, while in apple fiber, considerable amount of chlorogenic acid was found. From all these phenolic acids, ellagic and chlorogenic acids were detected in hydrogels. The type of pectin had an impact on the retention of ellagic acid, and a higher amount of this phenolic acid was detected in HMP hydrogels (5.6 mg/kg) regardless of fortification with apple fiber. While ellagic acid in hydrogels originated from blackberry juice, according to the results, chlorogenic acid originated from the apple fiber. Hydrogels prepared only with pectin did not contain chlorogenic acid, while in fortified hydrogels, this phenolic acid was determined. The higher amount of this phenolic acid was determined in LMP hydrogels (16.50 mg/kg). The same tendency was observed for rutin, i.e., it was determined in fortified hydrogels but in the same amount (20.4 mg/kg) regardless of pectin type. In addition to rutin, quercetin was detected in blackberry juice and was also determined in apple fibers. However, this flavonoid was not detected in hydrogels. In addition to quercetin in apple fibers, phloretin and phloridzin were also detected. Only phloridzin was determined in hydrogels fortified with apple fiber with a slightly higher amount in LMP hydrogels (95.29 mg/kg).

The stability of phenolic compounds over 8 months of storage was also determined and their retention after storage was calculated. The highest amount of cyanidin-3-*O*-glucoside was determined in fortified LMP hydrogel and HMP hydrogel (11.35 mg/kg) with retention rates of 19.9% and 16%, respectively, while the lowest retention of this anthocyanin was determined in fortified HMP hydrogel (12.9%). The amount of cyanidin-3-*O*-dioxalylglucoside was similar (around 7.10 mg/kg) in all hydrogels except for fortified HMP hydrogel (6.45 mg/kg). Even though this anthocyanin was detected in higher amounts in unfortified hydrogels, after storage its retention was higher in fortified ones, indicating that fortification of hydrogels with apple fiber caused its protection through storage. Its retention was 34.8% in fortified LMP hydrogel and 31% in fortified HMP hydrogel, while in the unfortified one, the retention was around 26.5%. Interestingly, ellagic acid was determined in a slightly higher amount after storage in both LMP hydrogels, probably due to the conversion of other phenolics into it (6.88 mg/kg and 7.98 mg/kg for unfortified and fortified one). In HMP hydrogels, retention of this phenolic acid was 86.3% and 83.5% for unfortified and fortified hydrogels, respectively. Chlorogenic acid was determined in a higher amount in fortified LMP hydrogel, and its retention was 87.6% while in HMP hydrogels, its retention was 79.7%. Rutin was identified only in fortified HMP hydrogel with retention of 62.3%. Phloridzin was detected in a much higher concentration in fortified LMP hydrogel (77.6 mg/kg) than in fortified HMP hydrogel (59.18 mg/kg) with a retention of 81.4% and 62.4%, respectively. Generally, it was concluded that after storage the highest retention of phenolic compounds was observed for fortified LMP hydrogels, except for rutin. By comparing the total concentrations of phenolic compounds, it was concluded that fortified hydrogels retained higher amounts of these valuable compounds than the unfortified ones.

Elevated temperature, as was the case for the preparation of hydrogels, can cause various reactions like oxidation, polymerization and/or condensation reactions of phenolics, leading to loss and/or formation of different compounds. Thermal degradation is one of the most important mechanisms of degradation of anthocyanins and it includes two main pathways [46]. One pathway includes the formation of a more unstable aglycon through hydrolysis of the 3-glycoside linkage. The other pathway includes the formation of a substituted chalcone through the hydrolytic opening of the pyrylium ring followed by its degradation to brown compounds of polyphenolic nature. The main anthocyanin in blackberry juice is cyanidin-3-*O*-glucoside. The initial reaction of its degradation in an aqueous solution starts with the hydrolysis of glucosidic bonds and the opening of the pyrylium ring due to heating [47]. The further step of degradation of this anthocyanin is the formation of protocatechuic acid and phloroglucinaldehyde. Furthermore, this anthocyanin can co-oxidize with other phenolics causing the formation of *O*-quinones that generate polymerized products by quinine–phenol reactions [48].

In our study, high retention of anthocyanins was achieved in unfortified hydrogels, while the addition of apple fibers caused a substantial degradation of these pigments. This can be explained by the pectin structure, which contributed to ionic interactions between anthocyanin cations and the carboxylic groups of pectin [49]. Higher retention of anthocyanins was observed by LMP hydrogels as low methoxylated pectin has a higher number of carboxylic groups. Consequently, these interactions may protect anthocyanins from a water attack avoiding the above-mentioned reactions, which leads to their stabilization [50]. Other studies have also proved that low methoxylated pectins have a higher positive effect on anthocyanins than high methoxylated pectin in raspberry jams, strawberry jams, and pectin model systems of black currant anthocyanins [32,33,34].

Generally, phenolics and dietary fibers can create chemical interactions in foods [51]. These interactions can occur via non-covalent bonds, such as hydrogen bonds (between oxygen atoms of the glycosidic bonds of polysaccharides and hydroxyl groups of polyphenols), hydrophobic interactions and van der Waals forces [52]. However, in our case addition of apple fiber caused a decrease in anthocyanins retention after preparation. Investigation of the impact of citrus fiber on the encapsulation of blackberry juice phenolics revealed that with the increase of citrus fiber amount, a decrease in adsorption of anthocyanins occurred [24]. Additionally, during the preparation of hydrogels, interactions between apple fibers and pectin were formed, causing reduction of binding sites for anthocyanins on pectin causing higher susceptibility of anthocyanins to a water attack. A positive effect of apple fibers was observed after storage that can be assigned to the co-pigmentation effect between phenolic compounds of apple fiber and anthocyanins, and between pectin of apple fiber and anthocyanins [34,50,53,54].

### 3.2. Antioxidant Activity

Antioxidant activities of blackberry juice, apple fibers and hydrogels (after preparation and after storage) are presented in Table 4. Comparing the results of antioxidant activities of blackberry juice and unfortified hydrogels, it was observed that the results of FRAP, CUPARC and ABTS methods showed that blackberry juice had higher antioxidant activities than hydrogels, while the results of the DPPH method showed the opposite effect. This contrasted with the expectation that all fortified hydrogels (due to higher total phenolics) would have higher antioxidant activities with all applied methods. For the FRAP, CUPRAC and DPPH methods, higher antioxidant activities of fortified hydrogels were observed than that for blackberry juice, while the ABTS results showed the opposite effect proving that the mechanism of action of determination of antioxidant activity is a very important factor. Comparing hydrogels, the results of the antioxidant activity determined with all four methods paralleled the results for total amounts of phenolic compounds i.e., pectin hydrogels had lower antioxidant activity than fortified hydrogels after preparation and after storage. After preparation, the application of the FRAP and CUPRAC methods showed that there was no difference between antioxidant activities of the LMP and HMP hydrogels, and both fortified hydrogels had the same values of antioxidant activities. However, a slightly different tendency was observed when the DPPH and ABTS methods were used. There was no difference between the LMP and HMP hydrogels, but fortified HMP hydrogel had higher antioxidant activities than fortified LMP hydrogel. After storage, a change of antioxidant activity occurred to a higher or lesser extent. Generally, the decrease of antioxidant activities after storage was not as pronounced as was the case with phenolic compounds.

Additionally, because blackberry juice and apple fiber possess antioxidant activity, the synergistic effects between phenolics in fortified hydrogels after preparation were evaluated. While the results for the DPPH and ABTS methods showed an antagonistic effect, for FRAP a slight antagonistic effect, for CUPRAC a quite high synergistic effect was observed. An antagonistic effect was expected as a high temperature was used during preparation causing various reactions on phenolics leading to loss and/or formation of different compounds with phenolic nature. However, these degradation products probably were responsible for the synergistic effect determined by the CUPRAC method. The results depended on the applied method [55,56]. Different behaviors could be explained based on the chemical nature and reactivity of the compounds formed. The radical scavenging capability of phenolics depends on their capability to donate hydrogen, thus a higher number of hydroxyl groups means a higher possibility of free radical scavenging activity. Availability of the hydroxyl groups is closely related to the chemical structure and spatial conformation; both factors can modify the reactivity of the molecules [57,58,59]. Some phenolics can have a strong tendency for polymerization reactions, which cause important structural changes, especially over time. The consequences of those reactions can be variations in the radical scavenging activity of the newly formed polymers. However, when the degree of polymerization exceeds a critical value, the increased molecular complexity of newly formed polymers can promote a decrease in antioxidant capacity due to the steric hindrance that can reduce the availability of the hydroxyl groups [57,60,61,62,63,64]. In addition to polymerization, other reactions on phenolics, like oxidation or degradation reactions can occur, also leading to a change in the antioxidant potential of compounds. Additionally, a very important aspect is the complexity of the matrix, i.e., the presence of other compounds and their structure. If there are other compounds present in the system, interactions can occur, also resulting in modification of the structure and availability of hydroxyl groups.

### 3.3. Inhibition of α-Glucosidase

α-glucosidase is an enzyme that is related to treatment of disorders connected with carbohydrate metabolism like hyperglycemia and diabetes [65]. In Table 5, concentrations of phenolic extracts that exhibited 50% inhibition of α-glucosidase activity (IC_50_ values) expressed as µg GAE/mL are presented. The results showed that all extracts had lower IC_50_ values than acarbose, which means that prepared samples were more effective than acarbose in the same assay system. The same effect was observed in the study by de Oliveira Raphaelli et al. [65], where apple phenolic extracts showed better inhibitory activity against α-glucosidase than acarbose. Minor modifications in assay conditions for determination of α-glucosidase inhibition make the results difficult to compare [50]. Except for prepared samples, blackberry juice and apple fiber were also analyzed for enzyme inhibition and the results showed that higher concentrations of phenolics from apple fiber were required to inhibit α-glucosidase by 50% (IC_50_ of blackberry juice was 0.87 µg GAE/mL and for apple fiber it was 22.23 µg GAE/mL). Blackberry juice showed the lowest IC_50_ value among all tested samples and could be used as a more potent inhibitor than acarbose. Samples after preparation and after storage showed the same trend, i.e., hydrogels fortified with apple fiber had higher IC_50_ values than those without apple fiber addition and ranged from 1.98 µg GAE/mL to 6.55 µg GAE/mL for samples after preparation, and from 3.86 µg GAE/mL to 10.47 µg GAE/mL for samples after storage. From the results obtained in this study, it was concluded that IC_50_ values of α-glucosidase inhibition after preparation depended on the concentration of individual phenolics, such as cyanidin-3-*O*-glucoside, rather than total phenolics determined spectrophotometrically. Higher concentration of this anthocyanin might cause lower IC_50_ values, which means that the lower concentration of this phenolic compound reduces the activity of α-glucosidase by 50%. It was previously reported that anthocyanins have a good potential for the prevention and treatment of diabetes mellitus [66,67]. Our results suggest that anthocyanins are good inhibitors of this enzyme. The same relationship between inhibition effectiveness and anthocyanin content in soft berry fruits was also observed elsewhere [68]. In the study of Xiao et al. [69], inhibitory effects of cyanidins were discussed and the following descending order of the activity against α-glucosidase was observed: cyanidin-3-sambubioside > cyanidin-3-glucoside > cyanidin. It was concluded that inhibitory activity was affected by glycosylation, which caused its increase, and by methylation, which caused its decrease. These results confirm previous knowledge about the relationship between polyphenol structure and enzyme activity [69]. Although other studies have reported an effective inhibition activity of phloretin toward glucosidase [70], in our case, apple fiber, where phloretin was present, possessed lower inhibition activity than blackberry juice. These beneficial effects of berry and apple fiber polyphenols may provide nutraceutical treatments for type II diabetes and obesity [71].

## 4. Conclusions

The results of this research showed the importance of proper formulation of the products. After the preparation of hydrogels, fortification of pectin/blackberry hydrogels with the apple fiber caused a decrease of anthocyanins content and α-glucosidase inhibitory activity, while on the other hand, an increase of total phenolics, proanthocyanidins and antioxidant potential was achieved. High retention of anthocyanins was achieved in unfortified hydrogels, while the addition of apple fibers caused a substantial degradation of these pigments, proving that interactions between compounds play important roles in retention of this valuable pigment, which also has antioxidant activity. On the other hand, those interaction cause formation of other phenolic compounds improving antioxidant potential of fortified hydrogels. Obtained hydrogels can be used as intermediate products or ingredients (like fruit fillings or spreads) for the improvement or development of novel food products to increase their fiber content and antioxidant potential.

## Figures and Tables

**Table 1:** Concentration for total phenolics (TPC) and proanthocyanidins (PAC) of hydrogels after preparation and after storage.

Sample	TPC (mg GAE/100 g)	PAC (µg/g)
blackberry juice	62.59 ± 0.63 ^a^	32.97 ± 0.29 ^a^
apple fiber	66.03 ± 1.78 ^b^	153.7 ± 1.7 ^b^
	Samples after preparation	
LMP	46.50 ± 0.95 ^a^	12.54 ± 0.18 ^a^
LMP + AF	97.60 ± 1.78 ^b^	98.55 ± 0.24 ^c^
HMP	43.30 ± 1.10 ^a^	14.66 ± 0.14 ^b^
HMP + AF	109.80 ± 0.07 ^c^	110.20 ± 0.41 ^d^
	Samples after storage	
LMP	45.10 ± 0.16 ^b^	7.93 ± 0.03 ^a^
LMP + AF	79.01 ± 0.94 ^d^	34.88 ± 0.18 ^d^
HMP	39.20 ± 0.10 ^a^	10.97 ± 0.06 ^b^
HMP + AF	75.8 ± 0.29 ^c^	28.42 ± 0.07 ^c^

LMP: low methoxylated pectin; HMP: high methoxylated pectin; AF: apple fiber. Within the column (separately for preparation and storage) means followed by different superscript letters are significantly different to each other at *p* ≤ 0.05 (ANOVA, Fisher’s LD).

**Table 2:** Individual phenolic compounds of blackberry juice and apple fiber determined by HPLC analysis.

Phenolic Compound	Blackberry Juice (mg/L)	Apple Fiber (mg/kg)
cyanidin-3-*O*-glucoside	75.38 ± 1.37	-
cyanidin-3-*O*-dioxalylglucoside	26.46 ± 0.33	-
ellagic acid	5.93 ± 0.15	-
chlorogenic acid	7.53 ± 0.12	51.64 ± 2.70
caffeic acid	0.82 ± 0.02	-
gallic acid	10.25 ± 0.08	-
rutin	1.23 ± 0.06	9.54 ± 1.14
quercetin	3.43 ± 0.02	130.60 ± 5.38
phloretin	-	17.64 ± 0.48
phloridzin	-	78.21 ± 0.60

**Table 3:** HPLC analysis of hydrogels after preparation (mg/kg) and after storage.

Hydrogels	c-3-g	c-3-dg	ea	cha	R	phl	*Total*
After preparation							
LMP	72.89 ± 0.19 ^d^	27.09 ± 0.13 ^c^	5.43 ± 0.02 ^a^	-	-	-	*105.41*
LMP + AF	57.15 ± 0.10 ^b^	20.56 ± 0.09 ^a^	5.44 ± 0.01 ^a^	16.50 ± 0.04 ^b^	20.37 ± 0.31 ^a^	95.29 ± 0.07 ^b^	*215.31*
HMP	70.50 ± 0.06 ^c^	26.01 ± 0.18 ^b^	5.71 ± 0.02 ^c^	-	-	-	*102.22*
HMP + AF	55.61 ± 0.03 ^a^	20.77 ± 0.41 ^a^	5.51 ± 0.01 ^b^	15.75 ± 0.04 ^a^	20.50 ± 0.09 ^a^	94.79 ± 0.01 ^a^	*212.93*
After storage							
LMP	10.46 ± 0.04 ^b^	7.03 ± 0.13 ^b^	6.88 ± 0.00 ^c^	-	-	-	*24.37*
LMP + AF	11.38 ± 0.17 ^c^	7.16 ± 0.09 ^b^	7.98 ± 0.00 ^d^	14.46 ± 1.12 ^b^	-	77.60 ± 0.17 ^b^	*118.58*
HMP	11.29 ± 0.03 ^c^	7.04 ± 0.00 ^b^	4.93 ± 0.04 ^b^	-	-	-	*23.26*
HMP + AF	7.19 ± 0.07 ^a^	6.45 ± 0.01 ^a^	4.60 ± 0.01 ^a^	12.56 ± 0.68 ^a^	12.78 ± 0.02 ^a^	59.18 ± 0.11 ^a^	*102.76*

LMP: low methoxylated pectin; HMP: high methoxylated pectin; AF: apple fiber; c-3-g: cyanidin-3-*O*-glucoside; c-3-dg: cyanidin-3-*O*-dioxalylglucoside; ea: ellagic acid; cha: chlorogenic acid; r: rutin; phl: phloridzin. Within the column (separately for preparation and storage) means followed by superscript different letters are significantly different at *p* ≤ 0.05 (ANOVA, Fisher’s LD).

**Table 4:** Antioxidant activities of blackberry juice, apple fibers and hydrogels (after preparation and after storage).

Sample	FRAP (µmol/100 g)	CUPRAC (µmol/100 g)	DPPH (µmol/100 g)	ABTS (µmol/100 g)
BJ	0.42 ± 0.01	23.36 ± 0.43	3.11 ± 0.02	4.47 ± 0.02
AF	0.42 ± 0.04	3.02 ± 0.01	4.17 ± 0.22	3.26 ± 0.10
*Theoretical AA*	*0.84*	*26.38*	*7.28*	*7.73*
LMP	0.33 ± 0.01 ^a^	18.97 ± 0.88 ^a^	3.88 ± 0.01 ^a^	1.39 ± 0.07 ^a^
LMP + AF	0.75 ± 0.01 ^b^	46.71 ± 0.31 ^b^	4.47 ± 0.08 ^b^	3.80 ± 0.02 ^b^
HMP	0.35 ± 0.01 ^a^	18.56 ± 0.56 ^a^	3.48 ± 0.33 ^a^	1.34 ± 0.01 ^a^
HMP + AF	0.77 ± 0.06 ^b^	47.98 ± 0.22 ^b^	4.86 ± 0.09 ^c^	4.03 ± 0.07 ^c^
Type of interaction for fortified hydrogels				
	Slight antagonistic	Synergistic	Antagonistic	Antagonistic
After storage				
LMP	0.31 ± 0.00 ^a^	15.43 ± 0.11 ^a^	2.68 ± 0.04 ^a^	1.07 ± 0.00 ^a^
LMP + AF	0.55 ± 0.00 ^c^	35.38 ± 0.56 ^c^	3.94 ± 0.04 ^b^	2.67 ± 0.02 ^c^
HMP	0.33 ± 0.00 ^a^	16.21 ± 0.83 ^a^	2.67 ± 0.04 ^a^	1.28 ± 0.02 ^b^
HMP + AF	0.45 ± 0.00 ^b^	28.06 ± 0.69 ^b^	3.66 ± 0.02 ^b^	1.92 ± 0.07 ^b^

LMP: low methoxylated pectin; HMP: high methoxylated pectin; AF: apple fiber; AA: antioxidant activity. Within the column (separately for preparation and storage) means followed by different superscript letters are significantly different to each other at *p* ≤ 0.05 (ANOVA, Fisher’s LD).

**Table 5:** Inhibition of α-glucosidase by acarbose, blackberry juice, and hydrogels (after preparation and after storage) expressed using IC_50_ (µg GAE/mL).

Inhibitor	IC_50_
Acarbose	105.61
Blackberry juice	0.87
Apple fiber	22.23
Samples after preparation	
LMP	1.98
LMP + AF	6.07
HMP	2.41
HMP + AF	6.55
Samples after storage	
LMP	3.86
LMP + AF	9.69
HMP	4.43
HMP + AF	10.47

LMP: low methoxylated pectin; HMP: high methoxylated pectin; AF: apple fiber.

## Data Availability

Data is contained within the article.
